# Measurement properties of the box and block test in children with unilateral cerebral palsy

**DOI:** 10.1038/s41598-021-00379-3

**Published:** 2021-10-25

**Authors:** Kai-Jie Liang, Hao-Ling Chen, Jeng-Yi Shieh, Tien-Ni Wang

**Affiliations:** 1 School of Occupational Therapy, College of Medicine, National Taiwan University, No.17, Xu-Zhou Rd. 4th Floor, Taipei City, 100 Taiwan; 2 Department of Physical Medicine and Rehabilitation, National Taiwan University Hospital, Taipei, 100 Taiwan

**Keywords:** Paediatric research, Neonatal brain damage, Outcomes research

## Abstract

This study aimed to examine the reliabilities (test–retest reliability and measurement error), construct validity, and the interpretability (minimal clinically important difference) of the Box and Block Test (BBT) to interpret test scores precisely for children with UCP. A total of 100 children with UCP were recruited and 50 children from the whole sample assessed the BBT twice within 2-week interval. The BBT, the Melbourne Assessment 2, the Bruininks–Oseretsky Test of Motor Proficiency, 2nd Edition, and the Pediatric Motor Activity Log Revised were measured before and immediately after a 36-h intensive neurorehabilitation intervention. Measurement properties of the BBT were performed according to the COnsensus-based Standards for the selection of health Measurement INstruments checklist. The test–retest reliability of the BBT was high (intraclass correlation coefficient = 0.98). The measurement error estimated by the MDC_95_ value was 5.95. Construct validity was considered good that 4 of 4 (100%) hypotheses were confirmed. The interpretability estimated by the MCID ranged from 5.29 to 6.46. The BBT is a reliable and valid tool for children with UCP. For research and clinical applications, an improvement of seven blocks on the BBT is recommended as an indicator of statistically significant and clinically important change.

## Introduction

Upper limb functional impairment is one of the most common problems in children with unilateral cerebral palsy (UCP)^1^. Such children tend to use their less-affected hand much more frequently than the more-affected hand, which can negatively affect the children’s motor development and further interfere with their participation in daily routines^2,3^. To decrease these limitations, clinicians dedicate considerable time and resources to facilitating their upper limb motor function^4,5^. Manual dexterity, an important indicator of upper limb motor function^6^, is frequently measured by clinicians and researchers to represent rehabilitative effectiveness^7,8^. Given that the improvement of dexterous function is a major goal of rehabilitative intervention, the use of an appropriate measure with sound psychometric properties is essential to ensure that the intervention outcomes can be measured accurately.

The Box and Block Test (BBT), developed by Mathiowetz, was designed to measure an individual’s manual dexterity^9^. It is a clinic-friendly standardized assessment that is portable, easy to obtain, simple to implement, and quick to administer without a specific environment. The BBT has been widely used as an outcome measure to present the effectiveness of upper limb rehabilitative programs in adult patients^1,10^. The psychometric properties of the BBT have been well established in adult populations, including patients with stroke, multiple sclerosis, and fibromyalgia^11–13^.

Recently, the BBT has also been commonly used in the pediatric field^7,14^. It is particularly suitable for children for several reasons. First, the evaluation method of the BBT examines essential components of manual dexterity for developing children, such as grasping, holding, transferring, and releasing. Second, the instructions of the BBT are simple to explain, and the task of the BBT is easy to understand. Third, it takes only one minute to administer the whole task, so it matches most children’s attention spans. Finally, it has been reported to be appropriate for repeated measurements as daily/weekly documentation for estimating the motor improvement curves of neurorehabilitation programs^14^.

The test–retest reliability, interrater reliability, and concurrent validity of the BBT have been investigated in typically developing children (TDC)^15^. The results indicate that the BBT demonstrates acceptable reliabilities (intraclass correlation coefficient, ICC = 0.85 –0.99) and is significantly correlated (*r* = 0.40–0.72 and 0.25–0.48 for age bands 1 and 2, respectively) with the manual dexterity subtest of the Movement Assessment Battery for Children–2 (MABC-2)^15^. Since the motor performance of children with UCP is very different from that of TDC, the reliabilities and validities from previous literature on TDC should not be extrapolated directly to children with UCP.

Although the BBT has been widely used to measure the effectiveness of neurorehabilitation programs in children with UCP^7,14,16,17^, only its test–retest reliability and responsiveness have been investigated^18^. The measurement error such as minimal detectable change (MDC, defined as the minimal amount of change that surpasses random measurement error)^19^, the construct validity, and the interpretability such as the minimal clinically important difference (MCID, defined as the minimal change score that is clinically meaningful for the respondents)^20^ of the BBT have not been investigated yet in children with UCP. For clinicians and researchers studying and treating upper limb impairments, an outcome measure with sound and comprehensive psychometric properties is indispensable to facilitate the interpretation and comparison of the results of controlled trials.

Therefore, the purpose of this study was to examine the psychometric properties of the BBT comprehensively, including the reliability, construct validity, and interpretability, in children with UCP. All properties of this study were in accordance with the guidelines of COnsensus-based Standards for the selection of health Measurement INstruments (COSMIN)^21^. The COSMIN is a standardized tool used to guide the studies on measurement properties.

## Results

The demographic characteristics are summarized in Table 1. The ICC of the BBT was 0.98 (95% CI = 0.96–0.99), indicating high test–retest reliability. The MDC_95_ value of the BBT was 5.95 (blocks) and the MDC% was 24%, showing acceptable random measurement error.

**Table 1:** Demographic and clinical characteristics of the participants.

Characteristic	Value				
Gender, female/male, n	49/51				
Age, mo, mean (SD)	102.59 (24.99)				
MACS, I/II**/**III**,** n	32/54/14				
More affected side, right/left, n	52/48				

Outcome measures	Pretreatment		Posttreatment		*p* value^a^
	Mean (SD)	95% CI	Mean (SD)	95% CI	
BBT	24.06 (12.92)	21.50–26.62	25.86 (14.34)	23.01–28.71	0.001
**MA2**					
Range of movement	20.20 (5.15)	19.16–21.23	20.85 (4.77)	19.88–21.81	0.002
Accuracy	21.45 (5.02)	20.43–22.46	22.35 (4.06)	21.53–23.18	< 0.001
Dexterity	10.06 (3.99)	9.26–10.87	10.62 (3.91)	9.83–11.41	0.002
Fluency	13.95 (3.28)	13.28–14.61	14.70 (3.45)	14.00–15.40	< 0.001
Subtest 3 of the BOT-2	19.14 (5.57)	18.02–20.27	20.19 (5.65)	19.05–21.32	< 0.001
**PMAL-R**					
AOU	1.93 (1.12)	1.70–2.16	2.35 (1.18)	2.11–2.59	< 0.001
QOM	2.28 (1.27)	2.04–2.55	2.63 (1.24)	2.39–2.89	< 0.001

*AOU* amount of use, *BBT* box and block test, *BOT-2* Bruininks–Oseretsky test of motor proficiency, 2nd edition, *MA2* Melbourne assessment 2, *MACS* the manual ability classification system, *PMAL-R* pediatric motor activity log-revised, *QOM* quality of movement.

^a^Paired-*t* test.

Four of the four hypotheses were confirmed to support the good construct validity of the BBT (Table 2). The interrelationships of the BBT and other selected measures were all statistically significant (p < 0.05; Table 3) at pretreatment and posttreatment. The score of the BBT had moderate to strong correlations with the four subtests of the MA2 (*r*s = 0.63–0.78, *p*s < 0.01), moderate correlations with the subtest 3 of the BOT-2 (*r*s = 0.49–0.57, *p*s < 0.01), and moderate correlations with the AOU/QOM of the PMAL-R (*r*s = 0.51–0.63, *p*s < 0.01). In addition, the results demonstrated that the correlation coefficients between the BBT and the MA2 were higher than those of the BBT and the other selected measures.

**Table 2:** Stated hypotheses and confirmed hypotheses for construct validity of the BBT.

Stated hypotheses	Expected correlations	Findings	Confirmed hypothesis
a. Positively strong correlation between dexterity subtest of the MA2 and the BBT	r > 0.7	r = 0.78	Yes
b. At least positively moderate correlations between the other subtests of the MA2 and the BBT	rs > 0.5	rs = 0.65–0.74	Yes
c. At least positively weak correlation between the BOT-2 and the BBT	r > 0.3	r = 0.49	Yes
d. At least positively weak correlations between the PMAL-R (AOU and QOM) and the BBT	r > 0.3	r = 0.51–0.54	Yes

*AOU* amount of use, *BBT* box and block test, *BOT-2* Bruininks–Oseretsky test of motor proficiency, 2nd edition, *MA2* Melbourne assessment 2, *PMAL-R* pediatric motor activity log-revised, *QOM* quality of movement.

**Table 3:** Correlations between the BBT and other measures at pretreatment and posttreatment.

	*r*^*a*^ (95% bootstrap confidence interval)	
	Pretreatment	Posttreatment
**MA2**		
Range of movement	0.74* (0.63–0.81)	0.72* (0.61–0.81)
Accuracy	0.72* (0.62–0.80)	0.68* (0.56–0.75)
Dexterity	0.78* (0.66–0.86)	0.76* (0.66–0.84)
Fluency	0.65* (0.51–0.76)	0.63* (0.50–0.74)
**BOT-2, subtest 3**	0.49* (0.34–0.62)	0.57* (0.43–0.69)
**PMAL-R**		
AOU	0.51* (0.34–0.64)	0.63* (0.48–0.75)
QOM	0.54* (0.38–0.68)	0.56* (0.38–0.71)

*AOU* amount of use, *BBT* box and block test, *BOT-2* Bruininks–Oseretsky test of motor proficiency, 2nd edition, *MA2* Melbourne assessment 2, *MACS* the manual ability classification system, *PMAL-R* pediatric motor activity log-revised, *QOM* quality of movement.

*P < 0.01.

^a^Validity values expressed as correlation coefficients (*r*) (95% bootstrap confidence interval).

For the interpretability, the distribution-based MCID of the BBT was 6.46 (Table 4). The anchor-based MCID was estimated as 5.29 (Table 4), based on children whose improvement scores of the QOM of the PMAL-R ranged from 0.38 to 0.74 points.

**Table 4:** Reliability and interpretability of the BBT.

	Reliability		Interpretability	
	Test–retest	Measurement error	MCID	
	ICC (95% CI)	MDC_95_ Score (%^a^)	Anchor (%^a^)	Distribution(%^a^)
BBT	0.98 (0.96–0.99)	5.95 (24)	5.29 (21%)	6.46 (26%)

*MCID* minimal clinically important difference, *MDC*_*95*_ minimal detectable change at 95% confidence.

^a^Proportion of the MDC_95_ or MCID score in mean of the BBT.

## Discussion

The findings of this study support that the BBT is a reliable, valid, and clinically applicable assessment that is adequate for measuring treatment outcomes in children with UCP. Regarding the test–retest reliability, the high ICC values of the BBT demonstrated that the BBT is a stable measure across a period of time. The high test–retest reliability is consistent with a previous study that used the BBT in children with CP (0.98 vs. 0.96)^18^. The MDC_95_ value can provide a useful benchmark to determine whether change scores surpass the measurement error. In our study, the MDC_95_ value of the BBT was 5.95, indicating that the performance of a child with UCP has to improve by more than 6 blocks after intervention for the change to be interpreted with a 95% confidence level as a true change. This finding was similar to that of a study by Chen et al.^22^, which reported that the measurement error of the BBT ranged from 5.5 to 7.8 blocks in patients with stroke. These MDC values can help clinicians to judge the significance of the results and to interpret the effectiveness of treatment^23^.

The construct validity of the BBT was good, as greater than 75% (100%) of the predefined hypotheses were confirmed. The correlation coefficients among the tests fluctuated only slightly between the pretreatment and posttreatment evaluations, suggesting that the relationships are relatively stable over different time frames. The BBT was moderately to strongly correlated with all subscales of the MA2, which measured quality of unilateral upper limb motor function in terms of range of movement, accuracy, dexterity and fluency. These results were in line with our expectation that manual dexterity (as measured by the BBT) would be correlated strongly with movement quality. In addition, moderate correlation between the BBT and subtest 3 of the BOT-2 was found. These findings indicated that the manual dexterity of the more-affected hand might reflect the bilateral motor performance of both hands to a moderate extent. The results of this study extend the validation study by Jongbloed-Pereboom et al.^15^, which examined the concurrent validity of the BBT in TDC. Furthermore, the correlation coefficients between the BBT and the bimanual motor tests were relatively more stable in children with UCP (*r*s = 0.49–0.57) than in TDC (*r*s = 0.40–0.72 for 3–6 years and 0.25–0.48 for 7–10 years)^15^, which supported our study rationale that psychometric properties obtained from TDC cannot be extrapolated directly to children with UCP.

The moderate correlations between the BBT and the PMAL-R, a parent-reported questionnaire, indicated that unilateral manual dexterity in children with UCP could partially reflect their parents’ perceptions of the child’s motor performance in daily contexts. These results also supported the previous finding that manual dexterity could be identified as an important attribute of the performance in daily activities^6^. Moreover, the correlations between the BBT and the MA2 (*r*s = 0.63–0.78) were relatively higher than those between the BBT and the PMAL-R (*r*s = 0.51–0.63). These findings accorded with our hypothesis that the relationships between the performance-based assessments would be stronger than those between the performance- and questionnaire-based assessments^24^. Overall, the findings of this study confirmed the BBT validly measures the construct we anticipated and indicated that the BBT can be used as an outcome measure for assessing upper limb motor function in children with UCP.

The MCID scores of this study were derived from an anchor, the PMAL-R, as well as from the distribution-based approach to represent the interpretability. In this study, the MCID estimate derived from the anchor reflected the participant’s perception of upper limb motor performance. The range of the MCID scores was 5.29 to 6.46, indicating that improvements of 5.29 to 6.46 blocks on the BBT could represent clinically meaningful change in daily motor activities. To compare the MDC and MCID estimates between different measurements, we calculated the MDC% and MCID% of the BBT. The MDC% (24%) and MCID% (21% to 26%) of the BBT were acceptable^25,26^, demonstrating the BBT is able to detect changes in clinical settings. However, the MDC% and MCID% of the BBT (21% to 26%) were somewhat higher than those of the MA2 (7% to 13%)^27^, indicating that children need larger improvements on the BBT to surpass the random error and to achieve the minimal clinically important difference. For individual-level interpretation, the MDC and MCID scores should be considered simultaneously^28^. It is reasonable to expect that the score of the MDC (measurement error) should be less than the score of the MCID (clinically meaningful change)^29^. Our findings showed that a child’s score needed to improve by 6 blocks to surpass the MDC value and by 7 blocks to surpass the MCID values. Therefore, if a child improves by 7 blocks on the BBT, it is likely to have clinically important change and the improvement is beyond measurement error. These indices are particularly useful for clinicians and researchers for interpreting the change scores precisely and accurately in children with UCP.

A few limitations of this study warrant consideration. First, the participants in this study were children with UCP with grasp capacity, so the generalization of our findings to children with other types of CP should be cautious. Further research should recruit more participants with other types of CP (e.g., dystonia and athetoid) or neurologic impairment to extend the application of the BBT. Second, we used the anchor from caregiver’s perspective (PMAL-R) to estimate the MCID instead of the subjectively described improvement from the participants. Choosing anchors from the viewpoint of participants such as Global Rating of Change scale could be established in future studies.

In conclusion, the BBT is a clinic-friendly standardized assessment and has been widely used to represent the effectiveness of upper limb interventions. The findings of this study confirm that the BBT has sound psychometric properties for measuring manual dexterity in children with UCP. For research and clinical applications, a minimum improvement of 7 blocks in the BBT can be interpreted as both statistically significant and clinically important.

## Methods

### Procedure and participants

The study procedure was divided into two stages. In the first stage, the participants were recruited through convenience sampling to estimate the test–retest reliability and the MDC until the target sample size (N = 50) was reached. The children were measured twice within one to two weeks before the neurorehabilitation intervention. In the second stage, a total of 100 children with UCP who finished the neurorehabilitation intervention and completed the pre- and post-treatment evaluations, 50 of whom were from the first stage, were included. All participants received a 36-h intensive neurorehabilitation program and were evaluated at pre- and posttreatment to estimate the construct validity and the values of MCID of the BBT. Participants could continue their usual rehabilitation care during the study period. The inclusion criteria were: (1) age of 5 to 12 years; (2) a diagnosis of spastic UCP; (3) no excessive muscle tone (Modified Ashworth Scale < 2 in upper limbs); (4) absence of severe cognitive, visual, or auditory disorders or involuntary movements leading to the inability to complete the measurement; and (5) no history of injections of botulinum toxin type A or operations on the upper extremity within 6 months. This study was approved by the Research Ethics Committee of the National Taiwan University Hospital (201512070RINA). Written informed assent/consent was obtained from the children and parents and all procedures were performed in accordance with relevant guidelines and regulations.

### Intervention

Eligible participants were assigned to receive the intensive upper limb neurorehabilitation program for a total training dosage of 36 h^30^. The intensive upper limb neurorehabilitation program was based on motor learning theory and emphasized the task-oriented approach^31,32^. The principles of shaping and repetitive task practice of upper limb movements were applied during the training sessions. Shaping is a training method in which a motor or behavioral objective is approached in small steps by successive approximations, and repetitive task practice involves functional tasks that are performed continuously over a specific period of time. The therapists graded the intervention tasks according to each child’s hand function and gave appropriate feedback to enhance motor learning. The tasks of each intervention protocol were chosen with consideration of the child’s specific upper limb impairments (e.g., reach, grasp, release, manipulate, etc.) and the appropriate level of difficulty, as well as the child’s preferences. The training activities were all provided by certified occupational therapists. Pre- and post-treatment assessments were administrated by the same rater, who was blind to the study design.

### Measurements

The BBT and three selected measures were used in this study: Melbourne Assessment 2 (MA2), Bruininks–Oseretsky Test of Motor Proficiency, 2nd Edition (BOT-2), and Pediatric Motor Activity Log Revised (PMAL-R). These measures (1) are frequently used in upper limb effectiveness studies in children with CP, and (2) have good psychometric properties for evaluating upper limb motor function^33,34^.

The BBT is a standard measure for evaluating manual dexterity^9^. In the administration the BBT, the participants grasp and transfer one-inch square blocks from one compartment to the other, transferring as many as possible. The number of blocks transferred from one side to the other within 1 min is recorded. Larger numbers of blocks correspond to better manual dexterity function. The MA2, which consists of 4 unidimensional subscales with 14 functional items, was used for measuring the quality of unilateral upper limb motor function. The 4 subscales, representing the 4 elements of upper limb movement quality, are range of movement, accuracy, dexterity and fluency^35^. The BOT-2 is a standardized assessment that is frequently used in upper limb neurorehabilitation effectiveness studies to measure bimanual coordination in children with UCP^36,37^. Subtest 3 of the BOT-2, manual dexterity, was used in this study. The PMAL-R is a questionnaire-based measurement completed by parents for assessing a child’s use of the more-affected hand in real-world situations^38^. It includes 22 tasks of daily living activities. How often (amount of use, AOU) and how well (quality of movement, QOM) the child uses the more-affected hand in daily life are measured. In summary, the MA2, the subtest 3 of the BOT-2, and the PMAL-R were used to estimate the construct validity of the BBT. Moreover, the QOM of the PMAL-R was used as an anchor to establish the MCID value of the BBT to reflect the subjective perception of improvement^39^.

### Statistical analysis

#### Estimation of the reliabilities

Test–retest reliability and the measurement error were used to describe reliability. The test–retest reliability was determined by calculating the ICC based on a two-way random-effects model at a 95% confidence interval (CI) and absolute agreement. Each participant was assessed twice within one to two weeks without additional intervention. The measurement error is defined as the systematic and random error of a participant’s score that is not attributed to true changes in the construct to be measured. The preferred and common statistic for measurement error in studies based on classical test theory is MDC^40^. The value of MDC represents the smallest amount of change beyond measurement error that reflects a score of true change^19^. It was calculated with a confidence level of 95% as follows: ${MDC}_{95}=1.96\times \sqrt{2}\times SEM=1.96\times SD\times \sqrt{2(1-ICC)}$, where SEM is standard error of the measurement, SD is standard deviation, and *ICC* is the coefficient of the test–retest reliability. Furthermore, to assess the extent of children’s changes after the intervention detected by the measurement, the MDC% was calculated by dividing the MDC by the scale width. For assessment that is absent of a ceiling score (e.g., the BBT), the mean score of the assessment from all observations was suggested as the alternate to replace the scale width^41^. The MDC% is independent of measurement units and can used to compare the magnitude of random measurement errors between assessments. An MDC% < 30% is considered to indicate acceptable random measurement error, and < 10% is excellent^25,26^.

#### Estimation of the construct validity

Construct validity is the degree to which the scores on a measurement are consistent with a priori formulated hypotheses based on the assumptions that the measurement validly measures a designate construct^21^. Good construct validity was determined as at least 75% of a priori hypotheses was confirmed^42^. Based on the COSMIN guideline, expected correlations with direction (positive or negative) and magnitude (absolute or relative) should be included in the hypotheses. These are the four hypotheses:

(a) Both dexterity subtest of the MA2 and the BBT measure similar construct. Thus, we hypothesized that the correlation between dexterity subtest of the MA2 and the BBT was positively strong.

(b) The BBT covers similar components of the motor abilities (e.g., grasping, holding, transferring, and releasing) as the other subtests of the MA2 (ROM, accuracy and fluency). At least positively moderate correlations were therefore hypothesized.

(c) Both the subtest 3 of the BOT-2 and the BBT asked a participant to perform the tasks in a limited time interval. However, the BOT-2 measure bimanual motor abilities, and the BBT measure unimanual motor abilities. Thus, we hypothesized that the correlation between the BOT-2 and the BBT should be at least positively weak.

(d) The correlations between observation-based and the questionnaire-based measurements are reported as weak to moderate^24^. We therefore hypothesized that the correlations between the BBT (observation-based) and the PMAL-R (questionnaire-based) should be at least positively weak.

Pearson correlation coefficients (r) were used by correlating the BBT with 3 selected measures (MA2, BOT-2, PMAL-R) at pretreatment and posttreatment. Strong correlations were defined as r ≥ 0.7, moderate correlations as 0.5–0.7, and weak correlations as 0.3–0.5^43^. To compare the relative magnitudes of correlation coefficients among the BBT with the 3 measures, 10,000 bootstrap samples computed with the percentile method were drawn from the dataset to estimate the 95% CIs of the correlation coefficients^44^. If the range of the 95% CI of a correlation coefficient did not contain the value of the other coefficient, it was considered to indicate a significant difference between the two coefficients.

#### Estimation of interpretability

Interpretability is the degree to which one can assign qualitative meaning (i.e., clinical connotations) to an instrument’s quantitative scores or change in scores^21^. Although interpretability is not categorized as a measurement property, it provides an important characteristic of a measurement instrument. Minimal (clinically) important difference (MCID) was used to describe interpretability of the BBT. Because there is no consensus on a standard method to determine the MCID, combinations of distribution- and anchor-based methods are recommended for triangulating a range of values for quantify the clinical importance^45^. The distribution-based method calculates MCID values from the data generated by the instrument itself by using the Cohen effect size benchmark. Effect size is defined as the difference in score from pre-treatment to post-treatment divided by the SD of the pre-treatment score. Half the SD of the pre-treatment score (to approximate Cohen’s moderate effect) of the BBT was used as the distributed-based MCID in this study^46^. The anchor-based approach of the MCID requires the identification of important degrees of improvement with an external standard. The PMAL-R QOM, a subjective questionnaire, was selected as the external standard to reflect the subjective perception of the children’s motor improvement. The anchor-based MCID was calculated as the mean change score of the BBT corresponding to participants who obtained the MCID scores on the PMAL-R QOM from pre-treatment to post-treatment. That is, children with improvements on the PMAL-R QOM of 0.38–0.74 were included in the calculation of the change scores of the BBT. The range of the PMAL-R MCID scores indicating that participants have subjectively experienced improvement was obtained from a previous study^39^. To verify whether the change of values was comparable between the BBT and other measurements, the MCID% was calculated by dividing the MCID by the mean score of the participants. Higher scores of the MCID% indicates the subject needs to make relatively large percentages of changes to achieve minimal clinically important difference.

## Abbreviations

AOU: Amount of use
BBT: Box and block test
BOT-2: Bruininks–Oseretsky test of motor proficiency, 2nd edition
ICC: Intraclass correlation coefficient
MACS: The manual ability classification system
MA2: Melbourne assessment 2
MDC: Minimal detectable change
MCID: Minimal clinically important difference
PMAL-R: Pediatric motor activity log-revised
QOM: Quality of movement
UCP: Unilateral cerebral palsy
